# Lifitegrast in Treatment of Dry Eye Disease—A Practical, Narrative Expert Review

**DOI:** 10.1155/joph/6504111

**Published:** 2025-01-16

**Authors:** Erlend C. S. Landsend, Mads Istre, Tor P. Utheim

**Affiliations:** ^1^Department of Ophthalmology, Oslo University Hospital, P.O. Box 4950, Nydalen, Oslo 0424, Norway; ^2^Department of Ear, Nose and Throat, Akershus University Hospital, P.O. Box 1000, Lørenskog 1478, Norway; ^3^Department of Medical Biochemistry, Oslo University Hospital, P.O. Box 4950, Nydalen, Oslo 0424, Norway; ^4^Department of Ophthalmology, Vestre Viken Hospital Trust, P.O. Box 800, Drammen 3004, Norway; ^5^Department of Ophthalmology, Vestfold Hospital Trust, P.O. Box 2168, Tønsberg 3103, Norway; ^6^Department of Ophthalmology, Sørlandet Hospital Trust, P.O. Box 416, Kristiansand 4604, Norway; ^7^The Norwegian Dry Eye Clinic, Ole Vigs Gate 32E, Oslo 0366, Norway

**Keywords:** cyclosporine, dry eye disease, inflammation, lifitegrast, neurons

## Abstract

**Purpose:** Dry eye disease (DED) is a multifactorial disorder affecting millions worldwide. Inflammation plays a central role in DED. The aim of this review is to critically evaluate the literature concerning the efficacy and safety of lifitegrast, a small molecule immunomodulator that blocks the action of lymphocyte function-associated antigen-1.

**Methods:** Studies were identified using PubMed and ClinicalTrials.gov. Fourteen studies met the inclusion criteria, six of which were randomized controlled trials. The articles were assessed regarding the effect of lifitegrast on symptoms and signs of DED, its usefulness compared to other treatments, and potential adverse events.

**Results:** The analysis demonstrated positive effects of lifitegrast on subjective and objective parameters of DED in the selected studies. However, despite promising results, the included studies did not provide enough evidence to conclude that lifitegrast could outperform other treatments of DED. No major side effects were reported.

**Conclusions:** Based on the current literature, we conclude that lifitegrast could improve various parameters of DED. Still, larger controlled trials are required to establish additional benefits of this medication beyond those of other DED treatments.

## 1. Introduction

Dry eye disease (DED) is defined as a multifactorial disease of the ocular surface, characterized by loss of the homeostasis of the tear film [1]. The disease causes symptoms such as pain and discomfort, but also reduced vision. DED is related to tear film instability and hyperosmolarity, inflammation and damage of the ocular surface, and abnormalities in sensory nerves [1, 2]. DED is highly prevalent [2, 3], and new treatments are sought to cope with the disease. Lifitegrast ophthalmic solution 5.0% (Xiidra, Shire, Lexington, MA, USA) is designed to inhibit ocular inflammation associated with DED [4]. As this treatment targets a central mechanism of DED, it has the potential to change the treatment pattern of DED. Hence, knowledge of lifitegrast is important for the ophthalmologist. The primary aim of the present review is to critically investigate the literature regarding treatment with lifitegrast eye drops, particularly studies reporting effects on signs and symptoms of DED. Studies comparing lifitegrast to a control group are considered especially interesting. In the review, we initially provide an introduction to the role of inflammation and ocular neurons in DED. From this background, the mechanisms of treatments targeting inflammation in DED are hopefully better understood. Various anti-inflammatory treatments are briefly described; however, with more detailed description of the action of lifitegrast. In the main body of this review, we evaluate 14 selected studies of treatment with lifitegrast and discuss their clinical significance.

Inflammation plays a central role in the pathophysiology of DED [5]. The inflammation could be initiated by increased osmolarity of the tear film, which is a hallmark of DED. Tear hyperosmolarity is preceded by either reduced tear production or increased tear evaporation. The hyperosmolarity starts a release of inflammatory substances that damage goblet and epithelial cells at the ocular surface. This damage may lead to tear film instability, early break-up of the tear film, and amplification of the tear hyperosmolarity—in a vicious circle.

**Figure 1:**
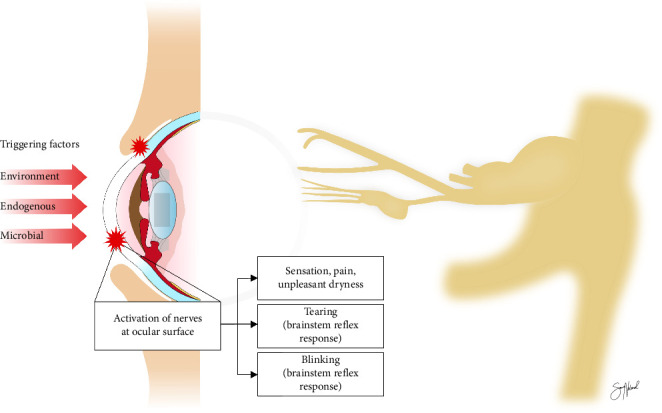
Peripheral and central neural mechanisms involved in the sensory and autonomic responses evoked by eye surface dryness. Triggering factors activate sensory neurons at the ocular surface (red stars). Signals from these neurons go mainly through branches of the ophthalmic nerve (highlighted in the figure). They pass further through the trigeminal ganglion and trigeminal nerve and cause symptoms such as pain and dryness. Signals are also transmitted through the brain stem and efferent pathways in a reflexive response causing tearing and blinking. Copyright: Sara Nøland.

Inflammation at the ocular surface probably activates peripheral sensory nerves, and leads to abnormal sensations and pain [6]. Inflammatory mediators could also give rise to nerve damage. Moreover, nerve injury could trigger an inflammatory reaction around the affected nerve axons. Consequently, progression of DED could be enhanced by an interaction between ocular sensory neurons and the local immune system. Inflammatory cytokines may also induce production of neurotrophic factors that stimulate nerve growth [7]. Potentially, these factors could cause abnormal nerve morphology, and further damage to the ocular surface. Neurons at the ocular surface induce impulses in response to triggering factors, which could be environmental, endogenous, and microbial, and influenced by genetic factors (Figure 1). Through a series of interneurons, these impulses affect sensation, tear flow, and blinking [6].

**Figure 2:**
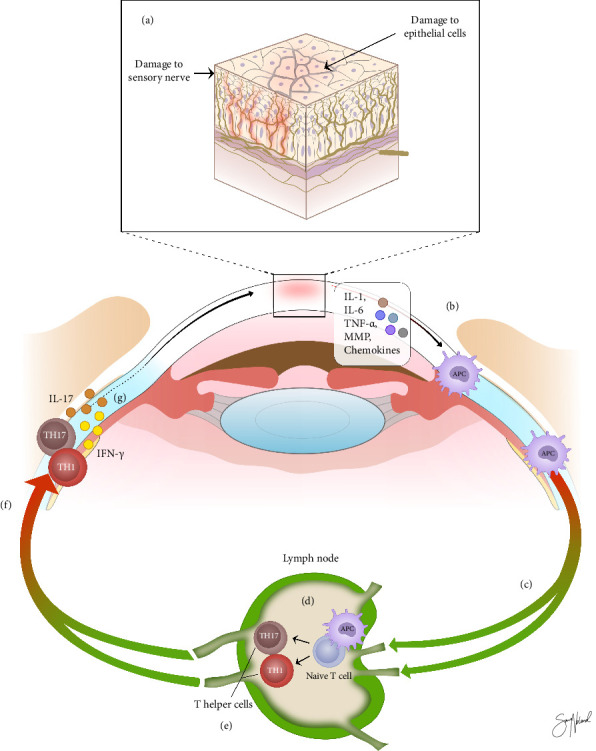
The immunoinflammatory pathway of dry eye disease. Trigger factors could cause damage to the ocular surface, including epithelial cells and sensory nerves in the cornea (a). In response, various proinflammatory mediators in the conjunctiva and corneal epithelium stimulate activation and maturation of APCs (b). APCs migrate to regional lymph nodes via afferent lymphatic vessels (c) and form an immunological synapse with naïve T-cells (d). Here, they make naïve T-cells ready to differentiate into different types of mature T-cells including the T helper cells T_H_1 and T_H_17 (e). These cells migrate through efferent blood vessels to the ocular surface (f), where the T_H_1 cells secrete the cytokine IFN-γ and the T_H_17 cells the cytokine IL-17 (g). APCs, antigen-presenting cells; IFN-γ, interferon gamma; IL, interleukin; MMP, matrix metalloproteinase; TNF-α, tumor necrosis factor alpha. Copyright: Sara Nøland.

Figure 2 illustrates the immunoinflammatory pathway of DED. Triggering factors at the ocular surface may create an immune response by releasing proinflammatory mediators [8]. These mediators include proinflammatory cytokines as interleukin (IL)-1, IL-6, and tumor necrosis factor-α (TNF-α). Further, matrix metalloproteinases (MMPs), and chemokines are involved [8]. The release of these proinflammatory substances induces maturation of antigen-presenting cells (APCs) localized in the ocular tissue. The mature APCs bear self-antigen and migrate to regional lymph nodes through afferent lymphatic vessels. Here, they make naive T-cells ready to differentiate into different types of mature T-cells including T helper cells and regulatory T-cells [9, 10]. The T helper cells T_H_1 and T_H_17 migrate through efferent blood vessels to the ocular surface [8–10]. T_H_1 cells secrete the cytokine interferon gamma (IFN-γ) that mediates goblet and epithelial cell death. T_H_17 cells secrete the cytokine IL-17 that promote corneal epithelial barrier disruption. Both IFN-γ and IL-17 amplify the cycle of inflammation by stimulating production of proinflammatory mediators, cell adhesion molecules, and factors inducing growth of lymphatic vessels [9]. For instance, IL-17 promotes secretion of MMPs from corneal epithelial cells and fibroblasts in response to hyperosmolar stress. MMPs are able to degrade the corneal epithelial barrier. The proinflammatory milieu also results in damage to corneal nerves, with nerve sprouts, tortuosity, and thinning commonly observed in DED [7]. This damage further intensifies the inflammatory cycle. Inflammation in DED can be treated with topical glucocorticoids, nonglucocorticoid immunomodulators, lymphocyte function-associated antigen 1 (LFA-1) antagonist, and systemic and topical antibiotics [11]. Nonglucocorticoid immunomodulators include cyclosporine A, tacrolimus, nonsteroidal anti-inflammatory drugs, biologic agents, and neuropeptides [11]. Treatment with antibiotics includes tetracycline and macrolide therapy.

The cyclosporine A eye drop is a nonglucocorticoid immunomodulatory medication that was approved for use in DED in 2003 [12]. Cyclosporine A inhibits production of IL-2 and activation of lymphocytes, and reduces apoptosis of epithelial and goblet cells [9]. Even though the clinical mechanisms are not fully clarified, cyclosporine A leads to increased tear production and increased goblet cell density [9]. Observational studies have shown some cases of long-term treatment-free remission of symptoms and signs of DED after treatment with topical cyclosporine A [13]. However, a Cochrane review concluded that the evidence on the effect of cyclosporine A on ocular discomfort and ocular surface parameters in DED is inconsistent. The parameters investigated were corneal fluorescein staining, Schirmer's test, and tear film break up time (TBUT), among others [14].

**Figure 3:**
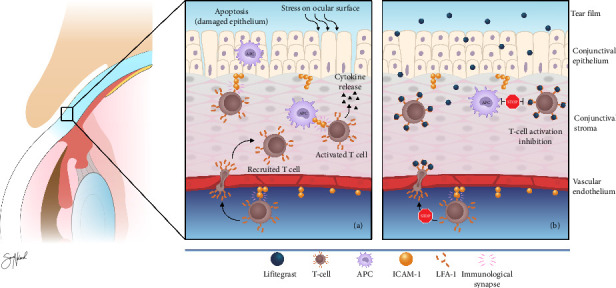
Mechanisms of action of lifitegrast. Binding of ICAM-1 to LFA-1 promotes recruitment and activation of T-cells in the conjunctiva (a). Lifitegrast blocks the binding of ICAM-1 to LFA-1 by connecting with the LFA-1 binding site (b). This blocking inhibits the recruitment and activation of T-cells (stop signs). APC, antigen-presenting cell; ICAM-1, intercellular adhesion molecule 1; LFA-1, lymphocyte function-associated antigen 1. Adapted from Periman et al. [9] Copyright: Sara Nøland.

Lifitegrast ophthalmic solution 5.0% (Xiidra, Shire, Lexington, MA, USA) is designed to inhibit ocular inflammation associated with DED [4]. It was approved for treatment of DED in the United States in July 2016. Lifitegrast blocks the function of LFA-1 [4, 15]. LFA-1 is an integrin, a transmembrane protein located on the surface of T-cells and other leukocytes [16]. This integrin modulates T-cell migration and activation (Figure 3) [10]. LFA-1 works through binding to the intercellular adhesion molecule 1 (ICAM-1). ICAM-1 is an adhesion protein that is expressed on several cell types, including APCs and endothelial cells [10, 16]. This protein is upregulated during inflammation. In DED, there may be increased expression of ICAM-1 on epithelial cells, activating T-cells in the corneal and conjunctival tissues.

The binding of ICAM-1 to LFA-1 promotes recruitment and activation of T-cells (Figure 3(a)). Lifitegrast inhibits the binding of ICAM-1 to LFA-1 on the T-cell surface by competitive binding to LFA-1 (Figure 3(b)) [10]. Through this action, lifitegrast blocks the following T-cell-mediated inflammation and averts the progression of an inflammatory response on the ocular surface. Lifitegrast has the possibility to influence both the afferent and efferent arm in the immunoinflammatory pathway of DED (Figure 2) [9]. This includes inhibiting migration of APCs to regional lymph nodes (afferent arm). In the efferent arm, lifitegrast may prevent migration, recruitment, and activation of T-cells in the ocular tissues.

## 2. Materials and Methods

**Figure 4:**
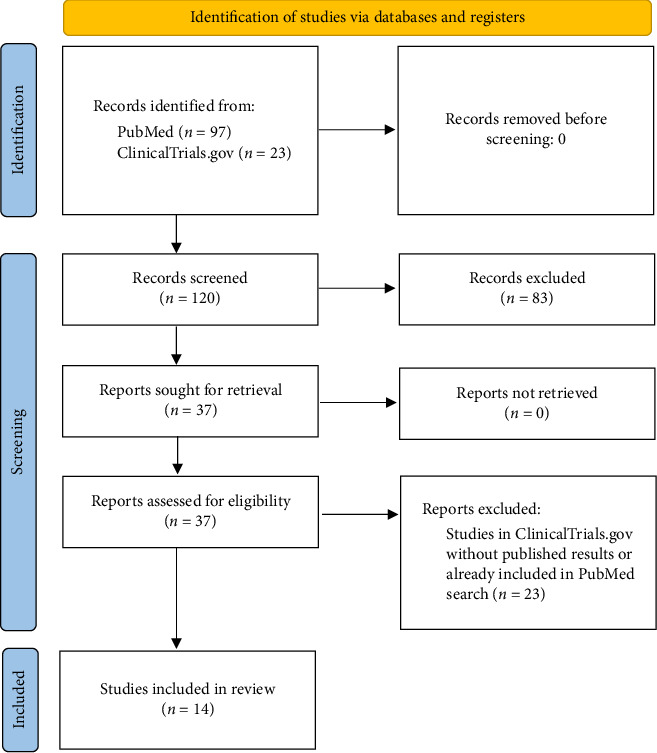
A modified PRISMA 2020 flow diagram showing results from the search and selection of literature. Based on: Page MJ, BMJ 2021; 372: n71. doi: 10.1136/bmj.n71.

A literature search was conducted in PubMed June 21^st^, 2020, using the words “Lifitegrast” and “Xiidra.” An updated search was performed in PubMed on January 24^th^, 2021, and January 10^th^, 2023, with the same search terms. The resulting articles were considered based on inclusion and exclusion criteria. Full text articles available in English were included in the review. Articles not written in English or only available as abstracts were excluded. Review articles were also excluded. Thus, only original papers concerning lifitegrast were finally included. A search was also performed in the clinical trial registry ClinicalTrials.gov with the same search terms as in PubMed. Only completed studies with fully published results were included from ClinicalTrials.gov. The methodology for search and selection of articles and studies are presented graphically in Figure 4.

## 3. Results

### 3.1. Results From Search and Selection of Literature

The initial search in PubMed gave 61 results, and the updated searches eight and 28 results, respectively. Thus, in total 97 publications from PubMed were considered. Based on the selection criteria, we finally included 14 of these articles in the present review (Figure 4). A summary of the included studies is presented in Table 1. The 14 studies comprised two cross-sectional surveys (CSS) [17, 18], four retrospective chart reviews (RCR) [19–22], two prospective longitudinal studies (PL) [23, 24], and six randomized controlled trials (RCT) (Table 1). Of the six RCTs, four investigated the efficacy and safety of lifitegrast compared to placebo [25–28]. One RCT evaluated the efficacy of lifitegrast versus thermal pulsation procedure (TPP) for treatment of meibomian gland dysfunction (MGD) [29]. The last of the RCTs, studied ocular comfort of lifitegrast, with placebo as control [15]. Five of the 14 reviewed papers were published in 2020 or later [17–20, 29].

All the studies considered both objective and subjective parameters, with a wide variation in the design of the trials. The number of patients included in the studies reporting patient outcomes varied from 14 [24] to 9772 [21]. In total, 12 clinical parameters were reported in the 14 studies. The most commonly reported parameter was symptoms (11 studies), followed by corneal staining (seven studies).  Table 2 gives an overview of changes in signs and symptoms presented in each article. One study (no. 6) [21], did not report patient outcomes, only adherence, and was therefore not included in Table 2. Differences in study design and reported parameters made direct comparison of the articles challenging. The most frequently reported outcome measure was symptoms. Four studies included both a group receiving lifitegrast and a control group receiving cyclosporine [17, 18, 20, 21], but only two of these studies included objective parameters [18, 20].

### 3.2. Effects on Subjective Scores

Scoring of symptoms varied between the reviewed articles. The only validated questionnaire reported was the Ocular Surface Disease Index (OSDI), which was used in four of the 14 articles [19, 24, 26, 28]. OSDI is a 12-item questionnaire designed to provide a quick assessment of the symptoms of ocular irritation consistent with DED [30]. Eight articles recorded improvement of DED symptoms with lifitegrast treatment, but without the use of validated questionnaires. Assessment tools in these studies included a drop comfort scale from 0 to 10 [15]; rating of treatment satisfaction, side effects, and limitation of activities [17, 18]; patient-reported DED symptoms [22]; an eye dryness score (0–100) [23, 25, 27]; or an eye discomfort scale [29]. One article did not report symptoms as an outcome at all [20].

The OSDI score improved significantly in patients receiving lifitegrast in three out of the four studies using this questionnaire [19, 24, 28]. The study without significant improvement in OSDI was a placebo controlled RCT [26]. This trial also used an ocular discomfort score and visual analogue scale as subjective measures of lifitegrast effectiveness. The visual analogue scale score improved after six and 12 weeks, and the ocular discomfort score after 12 weeks. Visual analogue scale is a 100-point scale. Zero implies no discomfort, whereas 100 points represents maximal discomfort of the following subjective measures: dryness, burning/stinging, photophobia, foreign body sensation, blurred vision, itching, and pain [26]. On the other hand, one of the studies showing significantly improved OSDI score did not demonstrate the same in ocular discomfort score or the visual analogue scale [28].

Using a visual analogue scale score, Tauber studied two groups, one receiving lifitegrast, and another receiving TPP [29]. The TPP procedure is designed to release the content of the meibomian glands by delivering heat to the inner eyelid surfaces while applying pulsating pressure to the outer eyelids. The symptom scores improved in both groups 6 weeks after treatment. However, dryness symptoms improved more in patients treated with lifitegrast than in those treated with TPP.

### 3.3. Effects on Objective Measures

The most commonly reported objective measures in the reviewed articles were amount of ocular surface staining (OSS), followed by TBUT, grading of MGD, concentration of MMP-9 (MMP-9) and Schirmer tear test (Table 2).

OSS is used as an overall term for all staining tests used in the reviewed studies. OSS was reported in eight studies (Table 2), but scoring of the staining varied between them. Corneal and conjunctival staining were reported in all these eight studies except for one [29], where only corneal staining was considered. Examples of tests included in the term OSS are corneal fluorescein staining score and conjunctival lissamine green staining score [26–28]. In patients that received lifitegrast, OSS decreased from baseline in six out of eight studies (Table 2). In one of these studies, the outcome measure was percentage of physicians considering lifitegrast to be successful at reducing OSS [18]. In the other five, reduction in OSS was calculated based on staining scores. One of the two studies not showing reduction in OSS, was a placebo-controlled RCT [27]. In this trial, there was no difference in inferior corneal staining, total corneal staining and nasal conjunctival lissamine staining between the lifitegrast and placebo group after 84 days of treatment. The other of the two studies, reported a trend towards improvement of corneal staining with lifitegrast treatment [23]. Comparing lifitegrast to TPP, Tabuer et al. found significant less OSS with lifitegrast compared to TPP following 42 days of treatment [29].

TBUT was assessed in four articles [18, 19, 23, 28]. Improvement in TBUT after treatment with lifitegrast was reported in two of these [18, 19]. Pepose, Qazi, and Devries showed a trend towards increased TBUT, without being statistically significant [23]. Semba et al. showed no statistically significant difference in TBUT between the lifitegrast and placebo group after treatment [28].

MGD grading was reported in three studies [19, 23, 29]. The grading was mainly based on the expressibility and quality of meibomian secretion (meibum). There was no statistically significant change in the parameters for MGD in either of the three studies. Tabuer showed a trend towards greater improvement in meibomian gland patency with lifitegrast treatment alone compared to TPP alone [29].

Concentration of MMP-9 was reported in three of the reviewed articles [19, 20, 29]. All these articles described significant reduction in MMP-9 levels after treatment with lifitegrast. Follow-up time varied between 6 weeks [29], 3 months [19], and 10.6 months [20]. In all three studies, MMP-9 was evaluated using a rapid, in-office test called InflammaDry (Quidel Corporation, San Diego, CA, USA) [31]. Any MMP-9 value ≥ 40 ng/mL was read as positive. Tauber also reported MMP-9 levels on a five point scale (0–4) based on intensity of color change of the test [29]. In this study, comparing treatment with lifitegrast and TPP, MMP-9 scores improved in both groups after 6 weeks.

Some other commonly used measures for DED did not consistently improve after lifitegrast treatment. This included tear osmolarity [23]. However, Pepose, Qazi, and Devries found a downward trend in tear osmolarity in patients responding to lifitegrast treatment [23]. Only one study reported tear debris [22]. After 6 months treatment with lifitegrast, tear debris improved in 74% of individuals presenting with this condition at baseline [22].

### 3.4. Comparison of Lifitegrast Versus Cyclosporine

In two CCS, Withe et al. assessed patient and physician experiences with current treatment of cyclosporine or lifitegrast [17, 18]. Most patients and physicians were satisfied with both cyclosporine and lifitegrast in reducing symptoms of DED. Though, limitations of both treatments included delayed onset of action, suboptimal symptom relief, and lack of improvement in patients quality of life [17, 18]. In the survey among physicians, 30 patients (14.3%) had been switched from lifitegrast to cyclosporine and 30 patients (14.3%) from cyclosporine to lifitegrast within the past 6 months [18]. In the survey among patients, 31 patients (15.0%) had shifted from lifitegrast to cyclosporine or other treatment, while 30 patients (14.5%) had shifted from cyclosporine to lifitegrast or other treatment within the past 6 months [17]. Despite switching, 37% of patients reported ineffective relief of symptoms. Onset of action and effectiveness after initiation of cyclosporine, and to a lesser extent lifitegrast, were the main reasons for change of therapy [17, 18]. Importantly, 36% and 31% of cyclosporine users and 45% and 40% of lifitegrast users had been on their current treatment for 6 months or less, in the two studies, respectively. In a nonrandomized open label clinical trial, Barber et al., reported that onset of action of cyclosporine could take up to 3–6 months [32]. On the other hand, Pepose, Qazi, and Devries showed that treatment with lifitegrast improved symptoms significantly already after 2 weeks [23].

Soifer et al. explored the level of MMP-9 in DED patients during treatment with either artificial tears, cyclosporine or lifitegrast [20]. Average follow up was 10.6 months. In this study, MMP-9 was evaluated using InflammaDry (Quidel Corporation, San Diego, CA, USA) bilaterally by the same trained technicians [31]. Patients were required to measure MMP-9 twice, at least 6 months apart. Only treatment with lifitegrast gave a statistical significant reduction in MMP-9 levels compared to artificial tears [20].

Treatment patterns of lifitegrast and cyclosporine among DED patients were reported in a retrospective cohort analysis [21]. Overall, 70.8% of patients treated with cyclosporine discontinued the treatment within median 89 days. In the lifitegrast group, 64.4% ended the intervention within median 29 days. The study did not register the reasons for discontinuation of treatment. However, financial considerations, human behavior regarding chronic medication use, the efficacy of treatment, or adverse side effect are mentioned as plausible causes [21].

### 3.5. Safety Profile

Adverse events were reported in all reviewed articles, except for three [20, 24, 29]. No serious adverse events were reported in any trial. Based on the available literature, lifitegrast treatment seems to be safe. Burning sensation, blurry vision, and dysgeusia were the most frequently reported side effects. Of those eight studies assessing side effects, five reported dysgeusia as the most common (prevalence varying from 12.9% to 29.0%) [17, 18, 22, 25, 27], while three studies found irritation upon installation of the drop as the most prevalent side effect (7.8%–24.0%) [15, 19, 26]. Sheppard et al., noted that lifitegrast gave irritation and discomfort primarily on the initial instilled dose that decreased over time [26]. In the study by White et al., dysgeusia was reported either “sometimes,” “usually,” or “always” by 56% of the patients receiving lifitegrast, but only in 21% of those receiving cyclosporine [17].

In the study by Atallah and co-authors, 10% of the participants receiving lifitegrast reported blurry vision at the post-treatment visits [22]. However, in the OPUS-3 phase III placebo-controlled trial, Nichols et al. did not find any change in best-corrected visual acuity after 84 days treatment with lifitegrast [s115]. This is consistent with findings in the OPUS-1 phase III study [26]. In this latter study, no significant changes were reported in intraocular pressure, corneal sensitivity, or dilated fundoscopy examination after treatment. In the OPUS-3 phase III trial, no indications of systemic adverse events were found, including no tendencies towards systemic toxicities, localized or systemic infections, or immunosuppressive complications [25]. Neither were any serious nonocular adverse events found in the phase II study of lifitegrast [28].

## 4. Discussion

### 4.1. Efficacy of Treatment

All of the included studies in this review reported improvement of at least one parameter for DED after treatment with lifitegrast. However, the few trials that did not compare lifitegrast with placebo or artificial tears failed to show a clear advantage over control groups. The exception was a PL comparing lifitegrast with TPP, which detected significantly better subjective and objective outcomes in lifitegrast-treated patients. Studies comparing lifitegrast with cyclosporine did not indicate superiority of lifitegrast over cyclosporine in treatment of DED. However, in one trial investigating MMP-9 levels with these two treatments, only the group receiving lifitegrast showed significantly reduced levels compared to artificial tears [20]. Interestingly, the authors did not find a significant relationship between normalization of MMP-9 and improvement of DED symptoms. Correspondingly, another study did not detect any difference in symptoms and signs of DED between those testing positive or negative for MMP-9 [33].

Although most physicians were satisfied with lifitegrast and cyclosporine for the treatment of DED, they agreed that more therapeutic options are needed [18]. There was a strong belief that these two medications are not sufficiently effective in managing symptoms or improving quality of life of patients with DED. The adherence to cyclosporine and lifitegrast was also low, with a high percentage of the patients discontinuing the treatment [21].

Taken together, this overview shows that there is evidence for the effectiveness of lifitegrast on subjective and objective parameters in DED. Nevertheless, studies with cyclosporine as control fail to show a clear additional benefit of lifitegrast [17, 18, 20]. On the other side, a meta-analysis found inconsistent evidence of the effect of cyclosporine A on ocular discomfort and ocular surface parameters [14]. Given that a sizable proportion of the patients in our review experienced significant improvement after treatment with lifitegrast, this may allude that lifitegrast could be more effective than cyclosporine.

### 4.2. Limitations of the Included Literature

A consistent limitation of the reviewed studies was relatively small sample sizes. There was also variation in calculation of sample sizes for statistical analysis. Further, the grading of symptoms, corneal staining, and MGD varied. For instance, several of the studies only presented self-reported symptoms that were either not quantified, or only quantified by using presence or absence of a specific symptom. That made direct comparison of the findings challenging. Similarly, the studies had a wide range in number of follow-ups and time between them. This limited comparison further and restricted the option to merge results across studies.

Another limitation of the assessed literature was lack of consistent inclusion criteria for the patients enrolled. Moreover, the current material included several retrospective or cross-sectional studies, with only one RCT comparing lifitegrast to other than a placebo group. In some of the studies the patients either received simultaneous treatment with other modalities or were not compared with control groups [22, 23].

### 4.3. Future Directions

Further studies into the long-term effects of lifitegrast are warranted to establish if this medication provides lasting improvement of symptoms and signs of DED. A core outcome set, which ideally includes both biomarkers and standardized patient‐reported outcomes in the field of DED, is needed.

With the current studies available, lifitegrast has not been shown to outperform cyclosporine. Further, it is currently not possible to conclude that lifitegrast is a sufficient treatment for DED. It is necessary to determine any long-term adverse effects of lifitegrast. Double-blinded studies investigating any possible symbiotic effects of lifitegrast with other treatment modalities such as local corticosteroids or antibiotics could also provide valuable insights.

## 5. Conclusions

Based on the current body of research, there is evidence for the effectiveness of lifitegrast on subjective and objective parameters of DED. Even though most studies report an improvement with lifitegrast, it has not yet been shown to consistently outperform cyclosporine in treatment of DED. Further studies on the potential added benefit of lifitegrast in the management of DED are required. Thus, this review highlights the need to design robust clinical trials that are double-blinded, randomized and controlled, with a sufficiently large sample size to draw reliable conclusions about this novel treatment.

## Figures and Tables

**Table 1:** Overview of key characteristics and findings of included articles, sorted by year published.

Study no.	First author	Year	Study objective	Participants follow-up (design)		Comments
1	Soifer et al. [20]	2021	Investigate correlation between MMP-9 and DED parameters	67 (RCR)	10.6 months	• Eyes with detectable MMP-9 had significantly lower tear production over time than those without detectable MMP-9 • Conversion to undetectable MMP-9 was more likely in eyes treated with lifitegrast than with artificial tears

2	White et al. [17]	2020	Investigate satisfaction among DED patients treated with LIF or CYC	207 (CSS)	None	• Satisfaction generally high with both LIF and CYC, however ineffective relieve of symptoms reported

3	White et al. [18]	2020	Investigate satisfaction among physicians treating DED patients with LIF and CYC	210 (CSS)	None	• After onset of action, 81% were satisfied with the effectiveness of CYC while 86% with LIF

4	Tong, Passi, and Gupta [19]	2020	Evaluate the effect and safety of LIF therapy in patients with DED	121 (RCR)	Average 88.1 days	• MMP-9 normalized in 38.9% of eyes after treatment. Significant improvement in ocular symptoms, corneal staining and tear film breakup time

5	Tauber [29]	2020	Compare the effect of LIF versus thermal pulsation procedure in meibomian gland dysfunction	50 (RCT)	42 days	• Eye dryness symptoms, corneal staining and eyelid redness improved more with LIF than thermal pulsation

6	White et al. [21]	2019	Evaluate the adherence, discontinuation, and switching of LIF and CYC in DED	9772 (RCR)	12 months	• Over 60% of the patients discontinued treatment within 12 months of initiation, within median 3 months for CYC and 1 month for LIF

7	Pepose, Qazi, and Devries [23]	2019	Test relation between tear osmolarity and DED symptoms in patients treated with LIF	26 (PL)	2, 6, and 12 weeks	• Significant improvement in symptoms after 2 weeks of treatment, which persisted throughout follow-up. Tear osmolarity did not predict reduction in symptoms

8	Atallah et al. [22]	2019	Evaluate benefits of 6 months LIF therapy in patients with DED	168 (RCR)	1, 2, 3, and 6 months	• Improvement of symptoms rated as significant by 56% of the participants and moderate by 36% • Signs of DED improved in most patients

9	Nichols et al. [15]	2018	Evaluate ocular comfort of LIF compared with placebo therapy in patients with DED	711 (RCT)	14, 42, and 84 days	• Drop comfort scores approached placebo levels 3 min after instillation with LIF, and was similar or better than in the placebo group at 5, 10, and 15 min postinstillation

10	Holland et al. [25]	2017	Investigate the efficacy and safety of LIF versus placebo in patients with DED	711 (RCT)	14, 42, and 84 days	• LIF improved DED symptoms significantly after 14, 42, and 84 days

11	De Paz, Gonzalez, and Ngo [24]	2017	Assess the effectiveness of LIF in reducing symptoms of eye dryness	14 (PL)	28 days	• OSDI improved significantly after LIF therapy • Subjects were allowed to use their current lubricating drops as needed during follow-up

12	Tauber et al. [27]	2015	To evaluate the efficacy and safety of LIF compared with placebo therapy in patients with DED	718 (RCT)	14, 42, and 84 days	• LIF significantly improved DED symptoms after 14, 42 and 84 days

13	Sheppard et al. [26]	2014	Assess the efficacy and safety of LIF compared with placebo in patients with DED	588 (RCT)	14, 42, and 84 days	• LIF significantly improved corneal fluorescein and conjunctival lissamine staining

14	Semba et al. [28]	2012	To evaluate the efficacy and safety of LIF compared with placebo in patients with DED	230 (RCT)	14, 42, and 84 days	• LIF significantly improved OSDI and corneal fluorescein staining

Abbreviations: CSS, cross-sectional survey; CYC, cyclosporine; DED, dry eye disease; LIF, lifitegrast treatment; MMP-9, matrix metalloproteinase-9 levels; OSDI, Ocular Surface Disease Index; PL, prospective longitudinal study; RCR, retrospective chart review; RCT, randomized controlled trial.

**Table 2:** Changes in signs and symptoms between baseline and last follow-up across studies.

No.	Year	Design	Symptoms	OSDI	TBUT	STT	MGD	MMP-9	OSS⁣^∗^	ER	TM	BCVA	TO	TD	Side effects
1	2021	RCR						↑^2 vs.⁣1^							
2	2020	CSS	↑^**2**^/↑^**3**^												+^2^/+^3^
3	2020	CSS	↑^**2**^/↑^**3**^		↑^**2**^/↑^**3**^	↑^**2**^/↑^**3**^			↑^**2**^/↑^**3**^						+^2^/+^3^
4	2020	RCR	↑	↑	↑		—	↑	↑						+
5	2020	RCT	↑^**2 vs. 4**^				(↑^**2 vs. 4**^)	↑^**2**^/↑^**4**^	↑^**2 vs. 4**^	↑^**2 vs. 4**^		—			
…															
7	2019	PL	↑		—		—		(↑)				—/(↑)		+
8	2019	RCR	↑						↑		—			↑	+
9	2018	RCT	(↑)												+
10	2017	RCT	↑^**2 vs. 5**^												+
11	2017	PL		↑											
12	2015	RCT	↑^**2 vs. 5**^						—						+
13	2014	RCT	↑^**2 vs. 5**^	—		—			↑^**2 vs. 5**^						+^2^/(+^5^)
14	2011	RCT	(↑^**2 vs. 5**^)	↑^**2 vs. 5**^	—	↑^**2 vs. 5**^			↑^**2 vs. 5**^						+

*Note:* ↑ = improvement, — = no difference, (↑) = trend towards improvement, + = side effects reported. Arrows and plusses without a number designate lifitegrast.

Abbreviations: BCVA, best-corrected visual acuity; CSS, cross-sectional survey; ER, eyelid redness; MGD, meibomian gland dysfunction grading; MMP-9, matrix metalloproteinase-9 levels; OSDI, Ocular Surface Disease Index; OSS⁣^∗^, ocular surface staining; PL, prospective longitudinal study; RCR, retrospective chart review, RCT, randomized controlled trial; STT, Schirmer tear test; TBUT, tear film breakup time; TD, tear debris; TM, tear meniscus level; TO, tear osmolarity.

^1^Artificial tears.

^2^Lifitegrast.

^3^Cyclosporine.

^4^Thermal pulsation procedure.

^5^Placebo/vehicle.

⁣^∗^OSS includes conjunctival and corneal staining scores, corneal fluorescein stain, conjunctival staining score, conjunctival staining (lissamine green), inferior corneal staining score, superficial punctate keratitis.

## Data Availability

The data analyzed in this study are included in the manuscript and the referenced articles.
